# Atmospheric oxygen and methane on the early Earth

**DOI:** 10.1098/rstb.2024.0093

**Published:** 2025-08-07

**Authors:** James F. Kasting, Aoshuang Ji

**Affiliations:** ^1^ Department of Geosciences, Penn State University, University Park, PA 16801, USA; ^2^ Department of Earth and Planetary Sciences, University of California, Riverside, CA 92521, USA

**Keywords:** atmospheric O_2_, atmospheric CH_4_, faint young Sun, greenhouse effect, glaciation, Snowball Earth

## Abstract

The habitability of the early Earth was affected by various factors. We focus here on atmospheric oxygen (O_2_) and methane (CH_4_). O_2_ is essential for supporting eukaryotic life, including plants and animals. CH_4_, along with CO_2_ and H_2_O, is a powerful greenhouse gas that may have helped offset the lower luminosity of the young Sun. Its concentration is disputed, though, in both the Archean and Proterozoic eons. The CH_4_ concentration depends strongly on atmospheric O_2_, which began to be produced biologically in geochemically significant amounts sometime during the Archean, probably near 2.7 Ga, based on the mass-independent sulphur isotope record and other (C and N) isotopic data. O_2_ was produced by cyanobacteria living in terrestrial mats and in the surface ocean during this time, generating localized oxygen oases that vented O_2_ into the atmosphere. Sometime between 2.2 and 2.4 Ga, a Great Oxidation Event occurred, causing O_2_ to increase substantially, likely to approximately 10 per cent of present levels. Increases in O_2_ near the beginning and end of the Proterozoic may have caused methane to decrease, triggering the Snowball Earth glaciations observed during both time intervals.

This article is part of the discussion meeting issue ‘Chance and purpose in the evolution of biospheres’.

## 1. Introduction

The habitability of early Earth was influenced by multiple factors, not all of which can be addressed in a short paper. The present manuscript will focus on atmospheric composition, specifically, the expected concentrations oxygen and methane during the Archean (3.8–2.5 Ga) and Proterozoic (2.5−0.6 Ga) aeons. Other important aspects of habitability, e.g. the faint young Sun problem, will be mentioned in passing, as they are intimately tied to the question of atmospheric composition and to the more general topic of the meeting for which this article was prepared. Those looking for more detail about the faint young Sun problem should consult reviews by Feulner [1] and Charnay and colleagues [2]. We will not discuss the preceding Hadean aeon (4.5−3.8 Ga), although it is equally important for habitability, as that is when life is thought to have originated. Readers interested in broader coverage of atmospheric and climate history should consult the book by Catling & Kasting [3].

The focus here will be on O_2_ and CH_4_ because these two gases are major exhaust products of organisms on the present Earth and probably on the early Earth as well. Atmospheric O_2_ levels are important because O_2_ affects the environment by oxidizing reduced gases and creating an ozone shield against solar UV radiation, as well as being required by eukaryotic organisms, including plants and animals, that derive their metabolic energy from respiration. Most authors agree that O_2_-producing cyanobacteria evolved sometime during the mid-to-late Archean [4], but the effects of this biological innovation on the Archean atmosphere are difficult to simulate accurately because cyanobacteria come in many different ‘flavours’, having different capabilities, and because O_2_ was not well mixed. One of our best geochemical indicators of atmospheric O_2_—mass-independent fractionation of sulphur isotopes (S-MIF)—is less useful than it might be because the causes of S-MIF are still poorly understood. Proterozoic O_2_ levels are also contentious, with estimates ranging from 10^−4^ PAL (times the present atmospheric level) [5] to 0.1 PAL [6]. We report on these topics in §2.

CH_4_ is a greenhouse gas with a present-day concentration of approximately 1.8 ppmv that is a significant contributor to modern, anthropogenic global warming. CH_4_ could have been much more abundant during the Archean when O_2_ levels were much lower because its lifetime would consequently have been much longer than today [7] and because the organisms that produce it—methanogens—are thought to be evolutionarily ancient [8]. Studies by our research group [9,10] suggest that Archean CH_4_ levels were much higher than today, at 100−1000 ppmv, which was enough to warm the climate by 6°−12° K [11]. These high values are supported by some independent models (e.g. [12]). But other authors argue that Archean CH_4_ levels could have been relatively low (1–50 ppmv) after the origin of oxygenic photosynthesis [13–15]. Curiously, Horne & Goldblatt [15] predict higher CH_4_ concentrations—approximately 100 ppmv—in the early- to mid-Proterozoic, in agreement with previous estimates by our group [16]. By contrast, Olson *et al.* [17] predict <10 ppmv CH_4_ at this time, and Laakso & Schrag [14] predict <1 ppmv. We re-examine the arguments for and against high Archean and Proterozoic CH_4_ levels in §3.

## 2. Evolution of atmospheric oxygen

### (a) Prebiotic/early postbiotic O_2_ levels

Any discussion of the rise of atmospheric O_2_ should begin with an analysis of O_2_ levels in the prebiotic atmosphere and/or in the postbiotic atmosphere prior to the origin of oxygenic photosynthesis. Prebiotic O_2_ levels were first studied by Berkner & Marshall back in the 1960s [18–21]. They assumed, correctly, that oxygen would be generated by the photolysis of H_2_O, followed by the escape of hydrogen to space. O_2_ and H_2_O absorb solar UV radiation at approximately the same wavelengths (<240 nm), so they reasoned that O_2_ would have accumulated until it shielded H_2_O from photolysis. H_2_O is largely confined to the troposphere by condensation, whereas O_2_ is not, so this reasoning seemed plausible at the time. This led them to predict prebiotic O_2_ levels of 10^−4^ to 10^−3^ PAL, which sounds low, but is well above the upper limit of 10^−5^ PAL necessary to explain the sulphur isotope signal recorded in Archean sediments (see §2b). So we know now that their analysis was incorrect.

The prebiotic O_2_ problem was re-examined by Walker [22]. Walker realized that O_2_ would have been consumed by reaction with reduced gases, e.g. H_2_, emitted from volcanos. He also understood that the H_2_ content of the atmosphere would have been determined by the balance between volcanic outgassing and loss to space, and that the escape rate of H_2_ could not have exceeded the so-called *diffusion limit*. The latter point is important because other authors, e.g. Brinkmann [23], had vastly overestimated the escape rate. The concept of limiting flux had been elucidated by Hunten [24,25] in a study that began with analysis of hydrogen escape from the atmosphere of Saturn’s moon, Titan. Based on this reasoning, Walker calculated a prebiotic atmospheric H_2_ mixing ratio of approximately 10^–3^.

In his 1977 book, Walker performed a back-of-the-envelope calculation that predicted a surface O_2_ number density of approximately 2 × 10^4^ molecules cm^−3^, which is <10^–14^ PAL. This value is more than 10 orders of magnitude less than what either Berkner and Marshall or Brinkmann had predicted, and it is still considered to be approximately correct. One can, however, improve on Walker’s estimate by doing a more detailed calculation with a one-dimensional photochemical model [26]. In doing so, one finds that the answer depends on the atmospheric CO_2_ level because O_2_ can be produced by reactions initiated by CO_2_ photolysis. Walker’s calculation had assumed present-day CO_2_ levels. For a CO_2_ concentration of 1000 PAL, consistent with values needed to offset lower solar luminosity, the calculated surface O_2_ mixing ratio is of the order of 10^−11^ PAL [27]. It should be remembered, however, that O_2_ is *not* well mixed in this type of weakly reduced atmosphere. The O_2_ mixing ratio in the upper atmosphere that is predicted by this same model is of the order of 10^−3^. So it is necessary to have spatial resolution—both vertical and horizontal—in one’s model if one wishes to study Archean O_2_ in any detail. We expand on this thought in §2*c*.

### (b) Geochemical indicators of O_2_: interpreting the sulphur MIF record

Much effort has been devoted to collecting and analysing geochemical data bearing on the rise of atmospheric O_2_. These studies were initiated many years ago by the Scottish scientist, Alexander MacGregor [28], who was studying banded iron-formations (BIFs). They were continued over the next century by many different workers, notably Cloud [29–31] and Holland [32–34]. Cloud and Holland studied a variety of O_2_ indicators, including BIFs, redbeds, palaeosols, detrital pyrite and uraninite, and fossil evidence for O_2_-requiring eukaryotes. We do not have space to review that evidence here, but it suggested to these authors that O_2_ first rose to appreciable levels sometime around 2 Ga, later revised to 2.2−2.4 Ga when more accurate age dates were obtained. Holland [34] termed this the ‘Great Oxidation Event’, or GOE, and that name has stuck since then.

**Figure 1.**
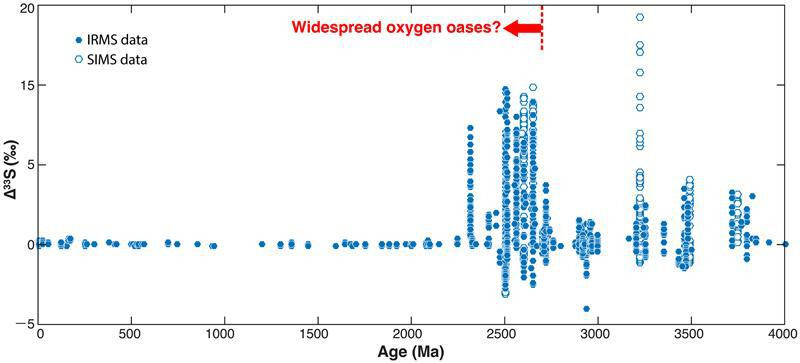
The sulphur MIF record, Δ^33^S, versus time. Modified from Ono [36]. The arrow indicating the presence of oxygen oases was added by us.

Disputes about the timing of the GOE continued throughout the 1990s because all the conventional O_2_ indicators were problematic in some sense, either because of difficulties in identifying them or because of complexities in their analysis. The debate was largely ended by the publication of a dataset showing mass-independent fractionation of sulphur isotopes back in the Archean [35]. An updated version of this dataset is shown in figure 1. The vertical axis shows Δ^33^S, a measure of the deviation of δ^33^S from the mass fractionation curve relating the two more abundant sulphur isotopes, ^32^S and ^34^S. (Sulphur has a fourth stable isotope, ^36^S, that is also useful for studying the rise of O_2_, but we will not be able to go into that detail here.) The S-MIF signal, as it is commonly called, is appreciable during the Archean, especially between 2.5 and 2.7 Ga, then goes away almost completely after about 2.3 Ga.

Since this dataset was first published, creative theoreticians have attempted to interpret it. Laboratory experiments showed that photolysis of gaseous SO_2_ produces mass-independently fractionated products, including sulphate and elemental sulphur (S_8_) [37]. Elemental sulphur was strongly enriched in ^33^S in these experiments, sulphate was either enriched or unfractionated (depending on the UV wavelength used) and residual SO_2_ was depleted. Because SO_2_ can be shielded by ozone, Farquhar *et al.* [37] interpreted the non-zero Δ^33^S values in their Archean dataset as having originated from SO_2_ photolysis in an anoxic, ozone-free atmosphere. This analysis agreed with the conventional indicators of the GOE tabulated by Cloud and Holland, so it has been widely accepted. Our own group performed calculations with our one-dimensional photochemical model that emphasized a different aspect of this problem [38]. At O_2_ levels above 10^−5^ PAL, virtually all SO_2_ emitted to the atmosphere by volcanoes is expected to be oxidized to sulphate, either in the atmosphere or in the surface ocean. This would homogenize all products of SO_2_ photolysis, thereby eliminating any S-MIF signal in the sedimentary record. So 10^−5^ PAL is commonly quoted as an upper limit on atmospheric O_2_ levels during the Archean, although, as we will argue below, the actual surface O_2_ levels were probably much lower.

In another sense, though, the debate about the meaning of the S-MIF data is far from over. Detailed photochemical modelling of SO_2_ photolysis in simulated Archean atmospheres by Claire *et al.* [39] and Izon *et al.* [40] failed to reproduce either the magnitude of the Archean S-MIF signal or the proper slope for the ratio Δ^36^S/Δ^33^S. (Mark Claire’s group did these calculations twice because the UV cross sections of the different SO_2_ isotopologues changed when they were remeasured.) Meanwhile, the quantum chemist, Dmitri Babikov, published a theoretical analysis [41] suggesting that the most important mechanism for producing S-MIF involves strong *negative* fractionation of ^33^S during formation of S_4_ and S_8_. This prediction appears at first glance to conflict with the study by Farquhar *et al.* [37] because the elemental sulphur produced in their experiments had *positive* Δ^33^S. But the laboratory results may have been influenced by self-shielding and/or by condensation of S_2_ on the walls of the reaction chamber [42,43]. Self-shielding occurs when the absorption bands of the major SO_2_ isotopologue, ^32^SO_2_, become saturated, so that only minor SO_2_ isotopologues are photolysed in the interior of the chamber. Unlike S_4_ and S_8_, which are both expected to be negatively fractionated in ^33^S by 500‰ during their formation, according to Babikov, S_2_ is expected to be unfractionated because of symmetry considerations. But self-shielding would overprint this signal, leaving condensed S_2_ with positive Δ^33^S.

Babikov’s chain-formation theory for producing S-MIF has been criticized by Goldman *et al.* [44]. These authors note that the effects of symmetry on chemical reaction rates have been studied on and off for several decades, and it has been argued that reactions such as those forming S_4_ and S_8_ should not fractionate isotopes in an anomalous fashion (see references in Goldman *et al*. [44]). We are encouraged, however, by recent experimental work on mass-independent fractionation of isotopes of Ti, O and Mg by Robert *et al.* [45,46]. They measured a significant MIF signal in reactions in which a solid precipitate is produced from a hot plasma. Their work is more relevant to the early solar system than to the Archean atmosphere; however, as pointed out by Endo *et al.* [47], it suggests that mass-independent fractionation during formation of a solid condensate from a gas mixture may be a universal phenomenon. We hope that someone can test Babikov’s chain formation theory in the laboratory.

### (c) Oxygen oases and their implications for sulphur MIF

By the beginning of the Archean aeon (3.8 Ga), life was likely present on Earth. The immediate effect on atmospheric O_2_ levels was probably minimal because the earliest forms of life, e.g. methanogens and anoxygenic photosynthetic bacteria, did not produce it. These anoxic ecosystems may have converted much of the available H_2_ to CH_4_, as discussed further below, but surface O_2_ levels should have remained essentially unchanged. There is, however, strong evidence that O_2_-producing cyanobacteria evolved sometime during the mid-to-late Archean. Geochemical evidence from Cr and Mo isotopes suggests that O_2_ was being produced as early as 3.0 Ga [5,48,49]. This timing is supported by molecular clock dating, which suggests that crown group cyanobacteria originated at 3.2 ± 0.3 Ga [50].

This does not necessarily imply that O_2_ production was widespread at 3.0 Ga. Nitrogen isotopes remain largely unfractionated from 3.2−2.8 Ga, suggesting an anaerobic marine environment during that time, despite some outliers in the 3.0 Ga Farrell Quartzite [51]. This pattern changes at approximately 2.7 Ga, when large N isotope fractionations are observed in multiple locations [52,53]. Evolutionary improvements in cyanobacterial fitness could be responsible for these changes. Might this be the time when they acquired the ability to fix nitrogen? Fixing N is not a trivial task for cyanobacteria, as the key enzyme needed, *nitrogenase,* is poisoned by O_2_.

Further evidence for increased O_2_ production at 2.7 Ga comes from S and C isotopes. The sulphur MIF record was shown in figure 1 and is discussed further below. Carbon isotopes in organic carbon (not shown) exhibit strongly negative ^13^C values (−50‰) at 2.7 Ga [54,55]. This exceeds the fractionation produced by any single carbon fixation mechanism. Recognizing this, Hayes [54] argued that this carbon must have been fractionated twice, once during its production, and then a second time when methane produced by its decay was consumed by methanotrophic bacteria that incorporated the carbon into their biomass. Methanotrophs are obligate aerobes, so this requires the presence of O_2_. Putative biomarker evidence for O_2_ production at 2.7 Ga [56,57] has now been discredited [58].

Observations like this lead to the concept of *oxygen oases*. This term was coined by Albert Fischer [59] in connection with cyanobacteria-containing microbial mats in which O_2_ may have accumulated, even though the overlying atmosphere was anoxic. It applies equally well to areas of the surface ocean where cyanobacterial blooms may have occurred during the post−2.7 Ga Archean. Escape of dissolved O_2_ to the atmosphere is limited by the so-called *piston velocity*, approximately 4 m d^−1^, which is the fastest rate at which O_2_ can diffuse through the uppermost boundary layer of the ocean. Kasting [60] used this concept to estimate a dissolved O_2_ concentration of 21 μM, or approximately 0.08 PAL, assuming O_2_ generation rates comparable to those today. A more sophisticated three-dimensional calculation using the GENIE biogeochemical model finds somewhat lower dissolved O_2_ concentrations of 1−10 μM [61]. A similar result is found in a spatially resolved box model calculation [13].

**Figure 2.**
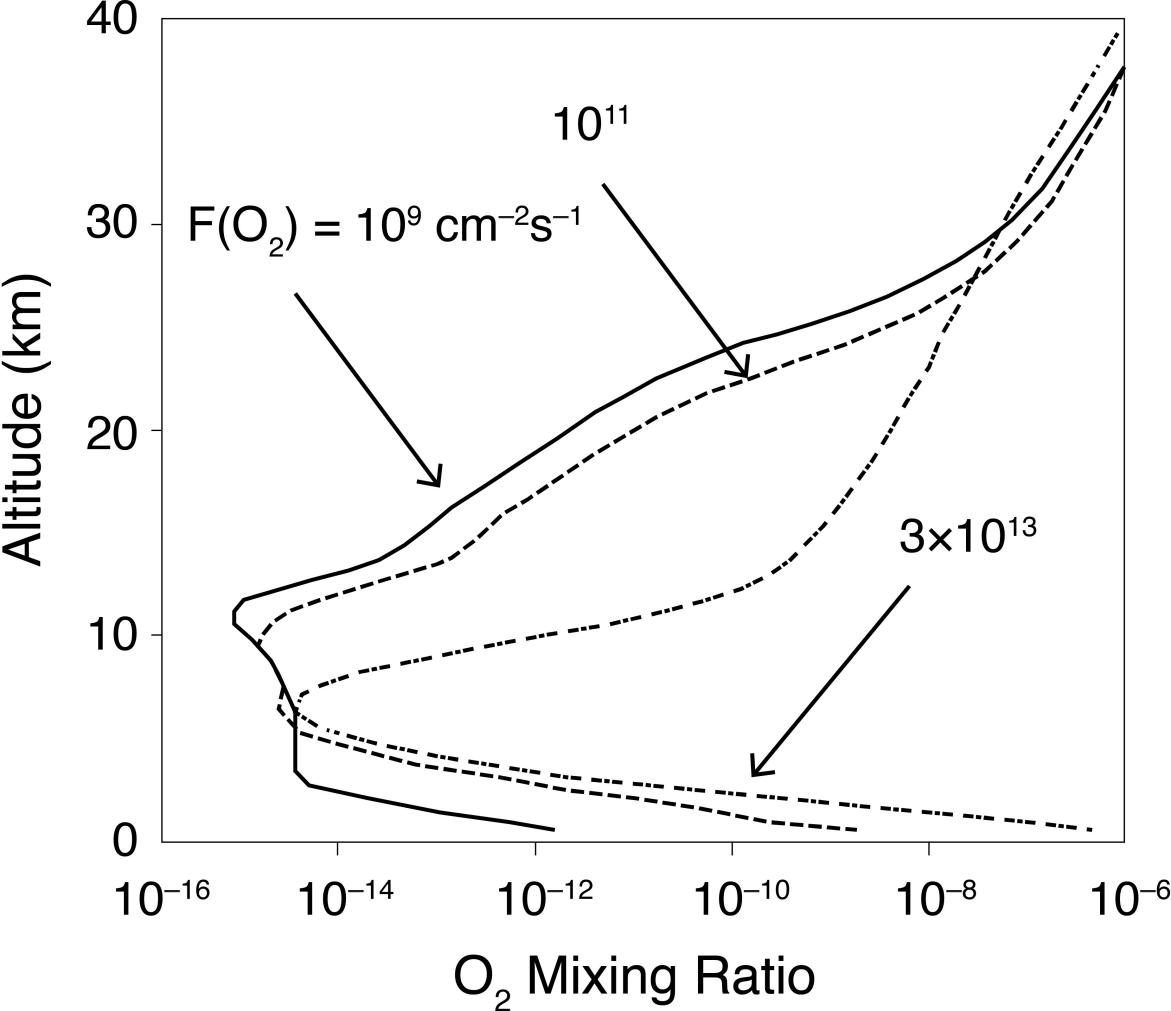
O_2_ mixing ratio vertical profiles calculated with a one-dimensional photochemical model for different assumed photosynthetic fluxes. The highest value 3×10^13^ cm^—2^ s^—1^shown corresponds to the modern, globally averaged, terrestrial plus marine flux (see text). From Pavlov *et al*. [9].

Simulating an Archean Earth containing oxygen oases with a one-dimensional model can lead to confusion because such models inherently assume that the atmosphere is horizontally well mixed, but that is unlikely to have been the case. Our own calculation from over 20 years ago [9] illustrates the problem (see figure 2). In this calculation, the background (1 bar, dominantly N_2_) atmosphere was assumed to contain 1000 ppmv CH_4_. O_2_ was injected into this atmosphere at various rates, simulating photosynthetic O_2_ release from the surface ocean. The highest rate shown, 3 × 10^13^ O_2_ molecules cm^–2^s^–1^, is close to the modern, marine plus terrestrial, rate of net primary production, 105 Gt(C) yr^–1^, which would generate approximately 9 × 10^9^ mol O_2_ yr^–1^, assuming standard photosynthetic stoichiometry: CO_2_ + H_2_O → CH_2_O + O_2_. (The conversion factor is 1 mol/yr = 3.74 × 10^–3^ molecules cm^–2^s^–1^.) The surface O_2_ mixing ratio reaches values as high as 10^–6^ in this calculation, but it drops rapidly with altitude. Pavlov *et al.* calculated surface O_2_ lifetimes of 1000−3000 s, or 0.3−1 h, in the absence of haze (see [9, figure 10b]). These can be checked by analyzing the steepness of the O_2_ decline above the surface in figure 2. In all cases, the O_2_ mixing ratio decreases by a factor of ~100 within the lowest 2 km. That is close to 5 e-foldings, so the characteristic e-folding distance, Δ*z*, is about 0.4 km, or 4 × 10^4^ cm. Vertical transport in the ne-dimensional model is modelled as eddy plus molecular diffusion, so the relevant transport time scale is (Δ*z*)^2^/*K*, where *K* (the eddy diffusion coefficient) = 10^5^ cm^2^s^–1^ in the troposphere. This yields an effective O_2_ lifetime of 1.6 × 10^4^ s, or about 4 h. The effective lifetime of O_2_ is somewhat longer than its photochemical lifetime because it can be transported in different forms, e.g. O_2_ and HO_2_. Still, this suggests that the O_2_ released from such oases should have formed plumes that dissipated as they drifted downwind, similar to pollution plumes in the modern atmosphere. Even three-dimensional models like GENIE are unable to simulate this behavior explicitly, as their spatial resolution is limited.

Let us now return to the sulphur MIF data shown in figure 1. Prior to 2.7 Ga, the MIF signal is muted, except for the sharp positive spike at approximately 3.3 Ga. But those latter datapoints represent individual pyrite grains measured by secondary ion mass spectroscopy, which might be expected to show a wider range of Δ^33^S values than bulk samples. The relatively small S-MIF signal seen between 3.2 and 2.8 Ga has been attributed to various causes: (i) an increased ratio of H_2_S to SO_2_ in volcanic gases [62], (ii) UV shielding of SO_2_ by organic haze [63,64], and/or (iii) the removal of most atmospheric sulphur as S_8_ [63,65]. In the latter two models, which assume the conventional isotopic fractionation mechanism [37], S_8_ has positive Δ^33^S, whereas sulphate and residual SO_2_ have negative Δ^33^S. Kurzweil *et al.* [65] attributed the large increase in fractionation seen from 2.7 to 2.5 Ga to the invention of oxygenic photosynthesis, which decreased the production of S_8_ and increased production of sulphate. This requires that the high-Δ^33^S datapoints shown in figure 1 come from elemental sulphur that was reduced to pyrite in the water column or in sediments. It may also require a late origin for sulphate-reducing bacteria, as the negative Δ^33^S values of pyrite formed from this process would otherwise dilute the positive values from S_8_ [62].

We agree with the basic suggestion of Zerkle *et al.* [63] and Kurzweil *et al.* [65], i.e. that increased O_2_ generation at 2.7 Ga caused the spike in positive Δ^33^S at that time. But the details of how this happened may be easier to explain using Babikov’s chain formation fractionation mechanism than with the conventional one. Increased O_2_ production at 2.7 Ga should have caused greater oxidative weathering of pyrite on the continents, speeding up the geochemical cycling of sulphur and thereby increasing release of sulphur gases by volcanos. This, in turn, should have led to increased photochemical *production* of S_8_, which varies approximately as the eighth power of sulphur gas input. (Note that this is just the opposite of the Zerkle/Kurzweil hypothesis, which was that S_8_ production *decreased* at this time.) In this alternative view, deposition of large amounts of S_8_ with negative Δ^33^S would have caused other atmospheric sulphur species, including SO_2_ and sulphate, to acquire strongly positive Δ^33^S. Spatial separation between the geographically limited oxygen oases and the anoxic remainder of the world would have minimized the problem of mixing of Δ^33^S signals from different reservoirs. And sulphate-reducing bacteria could have evolved early without diluting the positive Δ^33^S signals carried by SO_2_ and sulphate. So we think that Babikov’s proposed fractionation mechanism does a superior job of explaining the data shown in figure 1 [42].

### (d) Mid-Proterozoic O_2_ levels

Following the GOE, atmospheric O_2_ jumped up to appreciable levels, enough to make the S-MIF signal disappear. Debate continues, however, as to just how high those levels might have been. The ‘Boring Billion’ time interval from 1.8 to 0.8 Ga is perhaps the most interesting period, precisely because it was boring from the standpoint of both climate and carbon isotopes. This suggests that the Earth system somehow got stuck in a state that was quite different from the modern one. Virtually all workers agree that atmospheric O_2_ was lower than today, but estimates range approximately from 10^–4^ PAL to 0.1 PAL [49]. An accepted upper limit of 0.4 PAL comes from evidence for widespread anoxia in the ocean interior [66,67]. The lowest estimates come from data on chromium isotopes in shales and oolitic ironstones [5,68], cerium systematics in marine carbonates [69,70] and iron retention patterns in palaeosols [71] (but see Liu *et al.* [72] for a reanalysis of the Ce isotope data that predict pO_2_ > 1% PAL). In contrast, trace element abundances and isotopic signatures in shales [73–75] and iodine systematics of marine carbonate concretions [76] have been used to suggest higher atmospheric O_2_ levels, well above approximately 1% PAL and perhaps even >10% PAL.

**Figure 3.**
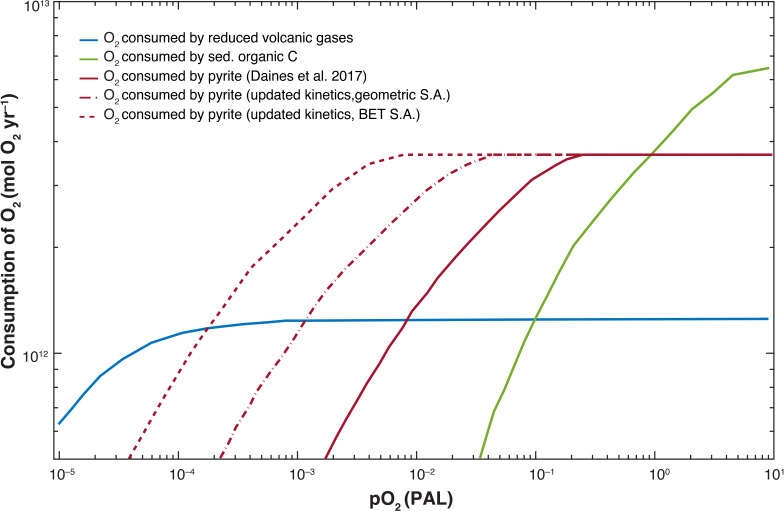
Global consumption rates of O_2_ by weathering of organic carbon (green, the rightmost solid) and pyrite (purple, the middle solid) and by oxidation of reduced volcanic gases (blue, the leftmost solid). Solid curves are from Daines *et al*. [6], who used the soil weathering model of Bolton *et al.* [77]. Dashed and dash-dot pyrite weathering curves are from Johnson *et al.* [78], who also used Bolton’s model but with different assumptions. The dash-dot curve assumes geometric particle surface area, while the dashed curve assumes surface area (S.A.) measured by the N_2_-BET (Brunauer–Emmett–Teller) method.

The problem with all these palaeo-O_2_ indicators is that they come either from marine environments, which can be out of equilibrium with the atmosphere, or from poorly characterized soil horizons. So models are needed to interpret these data, and not all models agree. Some models are better than others, though. A particularly good model, in our view, is that of Daines *et al.* [6]. These authors focus on the negative feedbacks necessary to stabilize O_2_ at different levels. Atmospheric O_2_ is generated by burial of reduced species in marine sediments, mostly organic carbon and pyrite, FeS_2_. It is consumed by oxidative weathering of organic matter and pyrite, oxidation of reduced volcanic and metamorphic gases and by seafloor serpentinization and oxidation. A list of estimated O_2_ sources and sinks on the modern Earth is shown in table 1. The O_2_ loss terms that Daines *et al.* consider in their model of the Proterozoic atmosphere are shown as a function of pO_2_ in figure 3. We have modified their figure by including two additional pyrite weathering curves calculated by Johnson *et al.* [78] using different assumptions about soil characteristics.

**Table 1.** Modern atmospheric O_2_ budget.

	rate (Tmol O_2_ yr^–1^)^a^
organic C burial	10.0 ± 1.7
pyrite burial	4.7 ± 1.2
Fe^2+^ burial	1.1 ± 0.6
total O_2_ production	15.8 ± 3.3
O_2_ sink	
organic C weathering	7.5 ± 1.7
pyrite weathering	3.5 ± 1.2
Fe^2+^ weathering	1.3 ± 0.4
surface volcanism	1.5 ± 0.7
submarine volcanism	0.7 ± 0.3
thermogenic/abiotic CH_4_	3.1
seafloor serpentinization, oxidation and Fe^2+^ input	0.4 ± 0.2
total O_2_ sinks	17.7 ± 3.5

^a^ Data taken from Catling and Kasting [3], tables 10.1–10.3. Data sources discussed in the accompanying text.

Today, Daines *et al.* [6] argue that stabilizing negative feedback in the oxygen cycle is provided by the (inhibitory) effect of O_2_ on the rate of organic carbon burial. That is because oxidation of organic carbon and pyrite during weathering is nearly complete on the modern Earth, according to the weathering model that they employed [77]. This model is represented by solid curves in figure 2. But at lower O_2_ levels, oxidation of both organic carbon and pyrite becomes incomplete. There is currently disagreement about pyrite oxidation rates. Daines *et al.* [6] suggest that pyrite weathering is only complete above approximately 0.1 PAL, whereas Johnson *et al.* [78] predict that pyrite oxidation could be complete at pO_2_ levels as low as 0.005 PAL. But, as Daines *et al.* [6] point out, this should not matter from the standpoint of atmospheric O_2_ stabilization because most of the sulphur entering the oceans is reburied as pyrite. The sulphur cycle can only provide negative feedback on pO_2_ if sulphur is weathered as pyrite and deposited as gypsum, CaSO_4_⋅2H_2_O. The numbers tabulated in table 1 suggest that pyrite burial exceeds pyrite oxidation today, indicating that the net effect of this cycle is to *produce* O_2_, not to consume it. Thick sulphate deposits are absent between 2.0 and 1.0 Ga [79], so pyrite weathering was not acting as a sink for O_2_ at that time, either. Daines *et al.* [6] neglect gypsum entirely for the Proterozoic, which is probably a safe assumption. So we agree with them that mid-Proterozoic atmospheric O_2_ levels were likely 0.1 PAL, or slightly above. If pO_2_ had dropped significantly below this level, consumption of O_2_ by oxidation of reduced volcanic gases would have exceeded consumption by weathering of organic carbon, and the atmosphere would have risked falling back into an anoxic, Archean-type state. Figure 1 indicates that such a backwards switch did not happen.

The analysis of Daines *et al.* [6] is supported by a study of petrographic carbon in mid-Proterozoic marine sediments, 742–1729 Ma [80]. The authors used Raman spectroscopy to look for organic carbon that had been recycled into sediments without being oxidized. They found little, suggesting that oxidation during weathering was essentially complete during that time. They then used the Bolton *et al.* [77] weathering model to calculate pO_2_ limits of 2–24% PAL. The upper limit came from assuming a modern continental uplift rate of 5 cm s^−1^, the same value as in Daines *et al*. [6]; the lower limit came from using an uplift rate that was 10 times lower. But using a lower uplift rate would not necessarily change the prediction that pO_2_ needed to be approximately 0.1 PAL to stabilize the oxygen budget: The green curve in figure 3 intersects the blue curve at that pO_2_ value, but it would really need to do so at a somewhat lower pO_2_ to ensure that the Proterozoic atmosphere remained consistently oxidizing.

Daines *et al*. [6] also pointed out that, according to their weathering rate curves, oxidation of pyrite would not have been complete until pO_2_ reached 0.1 PAL; thus, the absence of detrital pyrite in Proterozoic sediments is an additional argument for relatively high atmospheric O_2_ levels. This argument becomes weaker if the faster pyrite weathering curves of Johnson *et al.* [78] are correct. That said, other reduced minerals oxidize more slowly and may be used as a check. Notably, detrital uraninite (UO_2_) is observed today in the fast-flowing Indus River that drains the Himalayas [81], and one would expect this phenomenon to become more widespread if pO_2_ were significantly lower. So, the relatively high mid-Proterozoic O_2_ levels predicted by Daines *et al*. [6] are probably correct.

## 3. Atmospheric methane and its effect on climate

Let us return now to the question raised in §1 about atmospheric methane levels during the Archean and Proterozoic. Would they have been high enough to have had an appreciable warming effect on climate? We will not attempt to answer that question here, but we will examine the issues that must be resolved before such an answer can be given.

### (a) Archean methane levels

We begin with the Archean. In one sense, the question of early Archean methane is easy: methanogens evolved early, as pointed out in §1, and the lifetime of atmospheric methane in the pre-oxygenic photosynthesis atmosphere was 1000−10 000 years [7,9], compared with about 10 years today. So, even a relatively modest CH_4_ production rate like the present one—approximately 30 Tmol yr^–1^ [82]—could have produced CH_4_ concentrations of roughly 1000 ppmv.

Whether methane could have been produced at this rate during the Archean depends on the availability of hydrogen and the types of ecosystems that were present. Our own research group has produced a model [10] that predicts atmospheric CH_4_ levels ranging from 70 ppmv to almost 5000 ppmv, depending on the total hydrogen mixing ratio (see [10, table 2]). The total hydrogen mixing ratio depends on the volcanic outgassing rate and the escape rate of hydrogen to space, as discussed in §2*a*. The ecosystems investigated by Kharecha *et al.* [10] were all anaerobic. We mention two of them here: case 1 considered H_2_-using methanogens, acetogenic bacteria and acetotrophic methanogens, while case 3 included H_2_-using anoxygenic phototrophs, CO-consuming acetogens, acetogenic bacteria and acetotrophic methanogens. The organisms were not modelled explicitly; rather, the methanogens in case 1 were parameterized on the basis of how much thermodynamic free energy was needed to keep them going, whereas the anoxygenic phototrophs in case 3 were limited by the rate at which H_2_ could flow down through the boundary layer at the ocean surface.

Somewhat surprisingly, the case 1 and case 3 models yielded similar predictions for the concentration of atmospheric CH_4_, despite factors of 10−20 differences in calculated net primary productivity (NPP). The case 3 ecosystem was more productive, as one might expect, but the case 1 ecosystem produced just as much methane. This is because H_2_-utilizing methanogens produce approximately 10 CH_4_ molecules for every molecule of organic carbon that they incorporate into their bodies. As Kharecha *et al.* [10] pointed out, nutrient limitation of NPP by elements such as P and N should not have been a factor in the case 1 ecosystem—except perhaps at very high H_2_ outgassing rates [14]—-because the P requirements were minimal and because nitrogen fixation is widely accepted as being evolutionarily ancient [83]. Other modellers generally agree that an ecosystem in which primary productivity was dominated by H_2_-using (hydrogenotrophic) methanogens (case 1 of [10]) would have produced high CH_4_ concentrations (e.g. [12,14]). As the latter authors point out, the absence of photosynthesis allows hydrogenotrophs to consume most of the available nutrients. In their model, though, even the origin of anoxygenic, H_2_-based photosynthesis decreases the production of methane. The is because they assume a high organic burial fraction of 10–90% and have only a modest efficiency of 10% for acetotrophic methanogenesis, in which methane is produced by degradation of existing organic matter. By contrast, Ozaki *et al.* [12] continue to predict high CH_4_ concentrations (up to 3000 ppmv), particularly when Fe^2+^-based anoxygenic photosynthesis is added to the model biosphere. Their baseline organic burial fraction is only 2%, so nutrients are more available in their simulations.

Given the apparently early origin of cyanobacteria mentioned in §2*c*, the more interesting question is whether high methane levels persisted after the origin of oxygenic photosynthesis. This question was *not* addressed by Kharecha *et al.* [10]. Horne & Goldblatt [15] assumed that oxygenic photosynthesis had already originated by 3.5 Ga. In their model, the CH_4_ concentration dropped from approximately 150 ppmv to 50 ppmv at that time and then continued to decrease throughout the Archean, reaching 1 ppmv by the beginning of the GOE at 2.4 Ga. If that were the case then methane would have provided very little greenhouse warming in the late Archean. But their model did not calculate atmospheric H_2_, and hence it did not include H_2_-utilizing methanogens or H_2_-based anoxygenic phototrophs. If O_2_ was largely confined to the immediate vicinity of oxygen oases, as suggested in §2*c*, H_2_ could still have been widely available over much of the remaining planetary surface. A high-resolution, three-dimensional model would be needed to demonstrate this explicitly.

Laakso & Schrag [14] were even more pessimistic about the possibility of greenhouse warming by methane during the Archean. Like Horne and Goldblatt [15], they assumed an early date for the origin of oxygenic photosynthesis. Because the organic carbon burial fraction was high, nutrients were scarce in their model, and the CH_4_ mixing ratio never exceeded 1 ppmv during this time. Unlike Horne and Goldblatt, they *did* include an explicit H_2_ cycle and associated H_2_-utilizing metabolisms. But Archean primary productivity remained low in their model, so methane production was always limited. Their estimates for low productivity in the Archean oceans were based in part on ferruginous Lake Matano in Indonesia, which may not be representative of all ferruginous lakes (see §3b).

These low estimates for methane levels during the Archean fly in the face of the sulphur MIF data discussed in §2. Either CH_4_ or H_2_, or both gases, must have been abundant in the Archean to keep the atmosphere reduced. And if much of the Earth’s surface remained anoxic, it seems unlikely that methanogens would not have found a way to convert significant amounts of H_2_ to CH_4_? Independent evidence that H_2_ and/or CH_4_ *was* abundant during this time comes from Xe isotopes, which continued to be fractionated by escape of hydrogen in an ionized polar wind throughout this time [84]. The Zahnle *et al.* model [84] requires a total hydrogen mixing ratio (H_2_+2 CH_4_) of approximately 1% to carry off the xenon. In order to be balanced by volcanic outgassing, this may require that hydrogen was escaping at less than the diffusion-limited rate, but that seems entirely possible at these high total hydrogen concentrations.

### (b) Mid-Proterozoic methane levels

During the Proterozoic, pO_2_ should have been >10^–5^ PAL and the atmosphere should have been reasonably well mixed. But if the analysis presented in §2*d* is correct, O_2_ levels much below 0.1 PAL would have been unstable. For a mid-Proterozoic pO_2_ level of 0.1 PAL, the ozone UV screen should have been well established [85,86]. Low ozone column depths recently published by Cooke *et al.* [87] have been shown to be in error [88]. In the Ji *et al.* model [88], the atmospheric lifetime of CH_4_ at 0.1 PAL O_2_ is 50% longer than in the modern atmosphere (12 years versus 8 years, respectively), owing to a quirk of atmospheric chemistry: lightning produces less NO at lower pO_2_, leading to less production of CH_4_-destroying tropospheric ozone and OH. So, the question of whether CH_4_ levels were high during the mid-Proterozoic depends largely on its rate of production.

Pavlov *et al.* [16] estimated high methane levels of 100−300 ppmv during the Proterozoic, assuming that the organic carbon burial fraction was relatively low (2%), primary productivity was high and most of the organic matter produced was remineralized by fermentation and methanogenesis. The low burial fraction was based on data collected from the (sulphidic) modern Black Sea. In retrospect, all three assumptions are questionable, so their estimates may well be too high.

Horne & Goldblatt [15] also assumed a relatively low organic carbon burial fraction and found relatively high CH_4_ levels of 80−100 ppmv in their nominal model (see [15, figure 8]). But the Horne & Goldblatt model did not include a sulphur cycle, and hence it had no oceanic sulphate. The presence of sulphate decreases methane production both by reducing the amount of available organic matter in sediments (by sulphate reduction) and by enabling anaerobic oxidation of methane (AOM). So their model, too, may overestimate Proterozoic methane.

Olson *et al.* [17] used a three-dimensional, ocean-resolving geochemical model, GENIE, to estimate methane concentrations in the Proterozoic atmosphere. Their model *did* include oceanic sulphate, along with associated microbial metabolisms, i.e. sulphate reduction and AOM. Sulphate was specified in the surface ocean at levels ranging from zero to 2.8 mM (one-tenth of today’s value). CH_4_ oxidation in the atmosphere was parameterized following Goldblatt *et al.* [89]. Olson *et al.* [17] found that atmospheric CH_4_ was generally below 10 ppmv except at very low sulphate levels (<<1 mM) and relatively high pO_2_ (0.01−0.1 PAL). They calculated CH_4_ levels as high as 150 ppmv as sulphate dropped to zero. We have argued here that these high O_2_ levels are exactly what one should expect for the mid-Proterozoic. Whether surface ocean sulphate could have been below 1 mM remains to be determined. It should be noted that their model develops sulphate minimum zones in the deep ocean, analogous to today’s oxygen minimum zones, so some deep-sea sediments are completely devoid of sulphate reduction and AOM. Would these sulphate minimum zones spread even more widely if surface ocean sulphate were not fixed?

Finally, as discussed in the previous subsection, Laakso & Schrag [14] argued for high organic burial fractions and low methane throughout the Precambrian. They suggested that the ferruginous, low-sulphate, Lake Matano may be a good analogue for the Proterozoic oceans. Only approximately 10% of produced organic matter in Lake Matano is degraded by fermentation and methanogenesis, leaving the other 90% to be buried. If their analysis is correct, and if methane production from terrestrial ecosystems was not important during the Proterozoic, then both biogenic methane fluxes and atmospheric methane should have been limited during that time. Their upper limit on Proterozoic CH_4_ concentrations is 1 ppmv, which is the same as for the Archean.

Other ferruginous lakes, however, show lower organic carbon burial rates and higher methane production. Recent studies of Brownie Lake in Minnesota [90,91] suggest that 15%–38% of particulate organic carbon loading (POC) is deposited in sediments and 18 (± 7)% is converted to CH_4_. And these numbers are on the low end of the range of ferruginous lakes shown in

Akam *et al.* [91, figure 3], which have an average POC to CH_4_ conversion efficiency of 36% (± 21%). So these authors suggest that even under low primary productivity in Precambrian oceans, the efficient conversion of organic carbon to methane would have enabled marine CH_4_ to play a major role in early Earth’s biogeochemical evolution.

### (c) Implications for Precambrian climate

Finally, why should it matter whether Precambrian methane levels were high or low? High methane could, of course, have helped compensate for the faint young Sun. But, as discussed in §2, CO_2_ could have accomplished this task on its own, and the limited observational constraints from palaeosols do not rule this out. However, the controls on atmospheric CO_2_ operate relatively slowly and might be expected to have produced rather gentle, tectonically controlled changes in climate over time. By contrast, the Precambrian climate record shows evidence for six or more glaciations, including putative Snowball Earth episodes at 2.2−2.4 Ga and again at 0.8−0.6 Ga.

It is also noteworthy that the Snowball Earth episodes correspond closely to times when atmospheric O_2_ was increasing: the GOE at 2.2−2.4 Ga and the Neoproterozoic Oxidation Event (NOE) at 0.8−0.6 Ga [49]. If these global glaciations were triggered by decreases in CO_2_, then why should they be correlated with O_2_? But if they were triggered by decreases in CH_4_ then the correlation follows naturally, as one of us has pointed out repeatedly over the past 40 years (e.g. [16,92]). As both O_2_ and CH_4_ are both produced biologically, this suggests that life has indeed played an important role in Earth’s climate history, although its influence may have been generally destabilizing, rather than stabilizing.

## 4. Conclusions

Much is now understood about the evolution of atmospheric O_2_, but debate has continued concerning Mid-Proterozoic O_2_ levels and the mechanism(s) for producing Archean S-MIF. We argued here that Mid-Proterozoic O_2_ levels were relatively high, approximately 0.1 PAL, following logic presented originally by Daines *et al.* [6]. We also argued that the Archean S-MIF signal was mostly produced by the elemental sulphur chain formation mechanism proposed by Babikov [41]. This mechanism, coupled with localized oxygen oases in the Archean oceans following the expansion of oxygenic photosynthesis at approximately 2.7 Ga, can explain the basic features of the S-MIF record shown in figure 1. It must be confirmed by experiments, though, before it can be accepted.

Methane levels are widely believed to have been high, of the order of 100−1000 ppmv, during the early Archean, following the origin of life and prior to the origin of photosynthesis. This is sufficient to have provided 6−12°C of greenhouse warming, helping to offset the faint young Sun. That said, the main climate control throughout Earth’s history has probably been through CO_2_ and the negative feedback provided by the carbonate–silicate cycle. Methane levels may have decreased substantially following the onset of oxygenic photosynthesis, but this prediction deserves to be tested with models that account for the spatial isolation of oxygen oases and the persistence of high H_2_ levels elsewhere. Similar uncertainties surround the concentration of methane in the Mid-Proterozoic, with estimates ranging from <1 ppmv to ~100 ppmv. High levels of methane throughout the Archean and most of the Proterozoic could explain why Snowball Earth glaciations occurred near the ends of both eras.

## Data Availability

This article has no additional data.
